# One-Pot Synthesis of TiO_2_-rGO Photocatalysts for the Degradation of Groundwater Pollutants

**DOI:** 10.3390/ma14205938

**Published:** 2021-10-10

**Authors:** Stefano Andrea Balsamo, Roberto Fiorenza, Marcello Condorelli, Roberta Pecoraro, Maria Violetta Brundo, Francesca Lo Presti, Salvatore Sciré

**Affiliations:** 1 Department of Chemical Sciences, University of Catania, Viale A. Doria 6, 95125 Catania, Italy; stefano.balsamo@phd.unict.it (S.A.B.); marcello.condorelli@unict.it (M.C.); francesca.lopresti@phd.unict.it (F.L.P.); sscire@unict.it (S.S.); 2 Department of Biological, Geological and Environmental Science, University of Catania, Via Androne 81, 95124 Catania, Italy; roberta.pecoraro@unict.it (R.P.); mvbrundo@unict.it (M.V.B.)

**Keywords:** photocatalysis, titanium dioxide, reduced GO, 2,4-dichlorophenoxyacetic acid, water remediation

## Abstract

A non-conventional approach to prepare titanium dioxide-reduced graphene oxide (TiO_2_-rGO) nanocomposites based on solar photoreduction is here presented. The standard hydro-solvothermal synthesis of the TiO_2_-rGO composites requires high temperatures and several steps, whereas the proposed one-pot preparation allows one to obtain the photocatalysts with a simple and green procedure, by exploiting the photocatalytic properties of titania activated by the solar irradiation. The TiO_2_-rGO catalysts were tested in the solar photodegradation of a widely adopted toxic herbicide (2,4-Dichlorophenoxyacetic acid, 2,4-D), obtaining the 97% of degradation after 3 h of irradiation. The as-prepared TiO_2_-rGO composites were more active compared to the same photocatalysts prepared through the conventional thermal route. The structural, optical, and textural properties of the composites, determined by Raman, Photoluminescence, Fourier Transform InfraRed (FTIR), UV-vis diffuse reflectance (DRS) spectroscopies, and N_2_ absorption-desorption measurements, showed as the solar irradiation favors the reduction of graphene oxide with higher efficiency compared to the thermal-driven synthesis. Furthermore, the possible toxicity of the as-synthesized composites was measured exposing nauplii of microcrustacean *Artemia* sp. to solutions containing TiO_2_-rGO. The good results in the 2,4-D degradation process and the easiness of the TiO_2_-rGO synthesis allow to consider the proposed approach a promising strategy to obtain performing photocatalysts.

## 1. Introduction

The contamination of soil and groundwater is a critical environmental issue of our days [1,2]. Indeed, the high consumption of pesticides, as fungicides, insecticides, and herbicides, necessary to fulfill the growing current market demand, can generate serious problems for human and animal health. One of the widely used herbicides is the 2,4 dichlorophenoxiacetic acid (2,4-D), which has been on the market since 1945. The International Program on Chemical Safety (IPCS) classified it as a toxic and mutagenic agent. Moreover, in 2015 the World Health Organization (WHO), through the International Agency for Research on Cancer (IARC), has confirmed it as a carcinogenic contaminant [3,4]. For these reasons, it is important to develop new materials and strategies for the environmental remediation and water purification. By the way, among the advanced oxidation processes (AOPs), the photocatalysis is one of the most investigated, due to its good performance, biocompatibility, and low-cost implementation [5,6]. 

Titanium dioxide (TiO_2_) is the most employed semiconductor in this field, thanks to its good photocatalytic properties and easy preparation. However, two main drawbacks limit its use; the first one is the poor light absorption that is confined only in the UV region. This allows only a partial exploitation of the solar light that is fundamental for the sustainable large-scale applications. The second drawback is the fast charge recombination that decreases the photocatalytic activity of TiO_2_ [7,8,9]. Furthermore, the bandgap width is not the only critical parameter that influences the photocatalytic activity, being very important also the photocatalyst redox potentials and the related positions of the valence and conduction bands to generate the reactive radicals that drive the photodegradation pathway.

Several approaches have been investigated to overcome these limitations, including surface modification, doping, use of semiconductor or metal-based co-catalysts, and/or carbonaceous materials sensitization [10]. In particular, graphenic structures have emerged as the most promising materials that can be efficiently combined with TiO_2_, thanks to their excellent charge carrier mobility, acidic and basic inertness, good flexibility, and quite large specific surface area [11,12,13]. The features of these materials can strongly enhance the activity of titanium dioxide. Indeed, the good conductivity of graphene allows a strong interaction with the photogenerated electrons, extending the charge carriers lifetime and enhancing the photocatalytic activity. This is possible thanks to the Fermi level of graphenic materials that is below the conduction band of titanium dioxide [14]. Furthermore, some studies have highlighted how a wider light absorption, favored by the presence of the carbonaceous materials, leads to a remarkable increase of the photocatalytic performance of TiO_2_ [15,16,17,18,19]. The TiO_2_-rGO materials were also used for other environmental applications as gas sensors [18,19]. The peculiar features of GO and rGO that promote the charge carriers separation, their good affinity with TiO_2_, and the possibility to adopt easy and green strategies for their synthesis, has attracted a crescent interest for these materials compared to the others of the 2D family, especially in the field of the environmental protection.

However, it is difficult to obtain few layers of graphene, especially considering its great tendency to aggregate in polar solvent. This aspect is detrimental for the photocatalytic applications because an excessive formation of carbonaceous aggregates can lead to the surface coverage of the TiO_2_ active sites. A strategy to overcome this problem is the chemical functionalization of carbonaceous sheets in order to prevent their aggregation.

In the last ten years, several works report the use of reduced graphene oxide (rGO) coupled with titanium dioxide. rGO owns similar properties of the bare graphene, but its exfoliation is easier and the presence of some oxygenated residues facilitates the interaction with the titanium dioxide surface [20,21,22]. The most employed approach to reduce the GO is the thermal treatment. Indeed, it was widely reported that the C–O–C, the C=O, and the –COOH moieties of GO turn into C-OH bonds raising the temperature above 200 °C, thus obtaining the rGO with the restoration of part of the conjugated structure typical of bare graphene [23,24].

In 2008 *G. Williams* et al. [25] proposed a TiO_2_-assisted UV photoreduction of GO, exploiting the charge accumulation in a deaerated suspension of irradiated titania. In presence of a hole scavenger (h_scav_), the photoelectrons accumulated in titanium dioxide surface can reduce the GO following the reaction scheme:

TiO_2_ + hν(UV) → TiO_2_(h^+^ e^−^) (1)

TiO_2_(h^+^ e^−^) + h_scav_ → TiO_2_(e^−^)+ hscav^+^ (2)

TiO_2_(e^−^) + GO → TiO_2_ + rGO (3)

In this work, we have prepared TiO_2_-rGO composites through an alternative and green approach used for the GO reduction (i.e., the solar photoreduction), in order to avoid the high temperatures and pressures required for the standard hydrothermal synthesis [14]. Specifically, we have investigated for the first time, the photocatalytic activity of TiO_2_-rGO composites, obtained by the one-pot solar photoreduction of GO, in the photodegradation of an emerging contaminant of water as the 2,4-D pesticide. The high process versatility, which allowed for the use of the same photoreactor both for the materials preparation and for the photocatalytic tests, combined with the easy synthesis, can be a fascinating strategy to favor the scale-up of both processes (the synthesis of the TiO_2_-rGO samples and the photocatalytic wastewater treatment). Moreover, the use of the solar irradiation, instead of the UV light, is another attractive point that fits the today requests for a more intense use of renewables energies. The by-products formed during the photocatalytic oxidation have been investigated in order to check the safety of this process, whereas the eventual toxicity of the materials was examined with a short-term test employing nauplii of microcrustacean *Artemia* sp.

## 2. Materials and Methods

### 2.1. Samples Preparation

The samples were prepared starting from titanium dioxide Degussa P25 (TiO_2_) powders and graphene oxide (GO, purchased from Graphenea, San Sebastián, Spain) in water suspension (1 g/L). Briefly, 200 mg of TiO_2_ were suspended in 50 mL of ethanol in a batch pyrex jacketed reactor, and the suspension was then maintained under vigorous stirring. In addition, 4 mL of GO solution (in order to obtain the 2 wt.% GO) were sonicated for 30 min and added at the TiO_2_ suspension. This was purged with argon for two hours in order to remove all the oxygen present in solution and inside the reactor. After that, the solar radiation mimic lamp (OSRAM Vitalux 300 W, 300–2000 nm; OSRAM Opto Semiconductors GmbH, Leibniz, Regensburg Germany; solar irradiance: 10.7 mW/cm^2^, spectrum reported in the Figure S1) was turned on and the suspension was irradiated for 30 min at 25 °C. After centrifugation, the resulting powders were washed with water and left to dry in the oven at 70 °C for 24 h. The as-obtained sample was coded as TiO_2_-rGO solar. Another sample, named TiO_2_-rGO UV, was prepared with the same procedure described above, but irradiating with an UV lamp (Ted Pella, Redding, California, USA, UVP, 365 nm, 100 W). A third sample (TiO_2_-rGO thermal) was prepared in the same conditions without irradiation, calcining the powders at 230 °C in air, according with the first deoxygenation temperature reported in literature [23,24]. For comparison, the TiO_2_- unreduced GO sample was prepared in the same way, without the irradiation of the suspension and without thermal treatments, except for the drying at 70 °C for 24 h.

### 2.2. Samples Characterization

The textural properties of samples were analyzed by nitrogen adsorption-desorption measurements carried out with a Micromeritics Tristar II Plus 3020, (Micromeritics Instrument Corp. Norcross, USA) and using the Braunauer-Emmet-Teller (BET) and Barret, Joyner and Halenda (BJH) methodologies for the determination of the surface area and the pore size distribution, respectively. The samples were outgassed at 100 °C overnight. The morphological characterization was performed using a field emission scanning electron microscope (FE-SEM) ZEISS SUPRA 55 VP (Carl Zeiss QEC Gmb, Garching b. München, Germany). The optical properties were investigated by photoluminescence measurements performed with a Perkin Elmer LS45 (Perkin-Elmer, Waltham, MA, USA) with an excitation wavelength of 300 nm and by UV-vis DRS (Diffuse Reflectance spectroscopy) using a JASCO V-670 (Jasco Europe S.R.L., Cremella, Italy) equipped with an integration sphere and barium sulphate as reflectance standard. The optical band gaps were estimated according with the Kubelka-Munch theory [26,27]. However, it is important to point out that the estimation of energy gap with this methodology can lead to an inaccurate determination of the optical band gap. As reported in the literature, in fact, the reflectance spectra of the composites are more complex with respect to the pure oxides [28]. The structural properties were investigated through Fourier transform infrared spectroscopy (FTIR) using a Perkin-Elmer Spectrum Two FT-IR Spectrometer (Perkin-Elmer, Waltham, MA, USA) and by Raman spectroscopy using a WITec alpha 300 confocal Raman system (WITec Wissenschaftliche Instrument und Technologie GmbH Ulm, Germany) under the experimental conditions described in the ref. [29]. Zeta potential measurements were carried out with a HORIBA (Horiba UK Limited, Kyoto Close, Moulton ParkNorthamptonNN3 6FL, United Kingdom)Scientific nano particle analyser SZ-100 in disposable PMMA cuvettes with graphite electrode, applying a 3 mV potential.

### 2.3. Photocatalytic Tests

The photocatalytic experiments were performed using the same Pyrex batch reactor and the artificial solar irradiation described in the samples preparation. 50 mg of the photocatalyst were suspended in 50 mL of 2,4-D solution (5 × 10^−4^ M). The suspension was continuously stirred for two hours in the dark, to establish the adsorption-desorption equilibrium on the catalyst surface. Then, the lamp was turned on and aliquots (3 mL) of the suspension were withdrawn at constant time intervals of 30 min. The concentration of the contaminant was investigated with an UV-vis spectrophotometer (JASCO V-730, Jasco Europe S.R.L., Cremella, Italy) in Lambert-Beer regime, following the peak at 282 nm and reporting the C/C_0_ value as a function of time. C is the concentration at time t and C_0_ the starting value. In accordant with literature [30], the kinetic constants data were calculated considering, a first order reaction (Equation (4)):

k = −ln(C/C_0_) × t^−1^ (4)

Before the UV measurements, each aliquot was centrifuged for 20 min at 16,000 rpm in order to avoid scattering phenomena [31]. For the reusability tests, after each run the photocatalyst was filtered, centrifugated, washed with demineralized water, dried overnight, and re-used. The intermediate products formed during the photocatalytic tests were examined by ISI-MS (ion-spray ionization mass spectrometry) with an API 2000 instrument (AB Sciex Framingham, MA USA) equipped with a quadrupole detector. The TOC content was estimated with a TOC analyser (Shimadzu LCSH, Shimadzu Corporation, Kyoto, Japan) supplied by a non-dispersive IR detector (NDIR). The measure was carried out after a high-temperature treatment at 690 °C.

### 2.4. Toxicity Tests

*Artemia sp*. dehydrated cysts were used for the short-term toxicity tests. The durable cysts used in this study, marketed by JBL (JBL GmbH & Co. KG, Neuhofen, Germany), were hydrated with ASPM water to obtain nauplii (instar II-III larvae).

ASPM water is an artificial seawater solution made of different salts properly weighed (NaCl = 26.4 g, KCl = 0.84 g, CaCl_2_ × H_2_O= 1.67 g, MgCl × 6H_2_O = 4.6 g, MgSO_4_ × 7H_2_O = 5.58 g, NaHCO_3_ = 0.17 g, H_3_BO_3_ = 0.03 g), useful to guarantee the hatching of the cysts and to maintain nauplii on standard laboratory conditions (1.500 lux daylight; 26 ±1 °C).

The hatching of the cysts was carried out by immersing them in ASPM water inside a 1000 mL beaker and keeping them constantly oxygenated by an aerator for the insufflation of microbubble air: after 24–48 h, the hatching took place and the nauplii appeared.

Starting from a stock solution (1 mg rGO-TiO_2_ solar/10 mL ASPM water) of each type, we tested four different concentrations (serial dilutions ranging from 10^−1^ to 10^−4^ mg/mL). All the solutions have been sonicated (Bandelin, Sonopuls) for 12 min prior to use.

The nauplii in the second and third stages of development were collected one-to-one using stereomicroscope (Leica EZ4, Leica Microsystems Srl, Buccinasco (MI), Italy) and transferred into 96-well polystyrene microplates (BRANDplates^®^) where one nauplius per well was added. Every well has been quickly filled with 300 µL of each specific concentration of the solution used, and control samples were incubated only with ASPM water. Replication was performed for each concentration. Larvae were not fed during the exposure.

Finally, the microplates were placed on an orbital shaker and read after 24 and 48 h from inoculating the solutions to assess the endpoint (immobility/death) for the larvae by a stereomicroscope (Leica EZ4). Nauplii that appeared motionless were considered as dead, and the percentages of mortality were calculated for each treatment; if a larva does not move its antennae after agitation of the water for 10 s, it is counted as dead.

Statistical analysis was carried out by one-way ANOVA test to analyze differences between the controls and treated where p<0.05 is considered significant and *p* < 0.01 extremely significant.

## 3. Results and Discussion

### 3.1. Materials Characterization

Table 1 summarizes the textural properties of the samples determined by the nitrogen adsorption-desorption measurements. The N_2_ isotherms are reported in supplementary materials (Figures S2 and S3). There are no significant variations in the BET surface area, except for the TiO_2_-rGO thermal sample, which showed a lower value (44 m^2^·g^−1^) compared to the other two materials containing the rGO (55 and 53 m^2^·g^−1^ for TiO_2_-rGO UV and TiO_2_-rGO solar, respectively). The lower surface area of the TiO_2_-rGO thermal composite is probably due to the aggregation of the titanium dioxide nanoparticles favored by thermal treatments [32]. This is confirmed by the increase of mean pores diameter up to 18 nm for TiO_2_-rGO thermal, higher than the other two photo-reduced samples that exhibited values of 6 nm (TiO_2_-rGO solar) and 5 nm (TiO_2_-rGO UV). According to the literature, the photoreduction of GO leads to a decrease of the mean pores diameter [33].

Interestingly, the formation of rGO leads also to a change in the pores’ shape. In fact, all the samples showed the typical IV isotherms of mesoporous materials (Figures S2 and S3), whereas examining the hysteresis loop, it is possible to note that the TiO_2_ and TiO_2_-GO showed a H1 hysteresis related to uniform agglomerated spherical pores whereas the TiO_2_-rGO photo-reduced based composites exhibited a H2 hysteresis (Figures S2 and S3), typical of ink-bottle pores with thin necks and large bodies [34]. Finally, the thermal sample exhibited a H3 hysteresis, associated to agglomerates formed after the treatment [34] (Figure S3, Table 1).

The SEM images of the as-synthesized materials are reported in the supporting information (Figure S6). 

**Figure 1:**
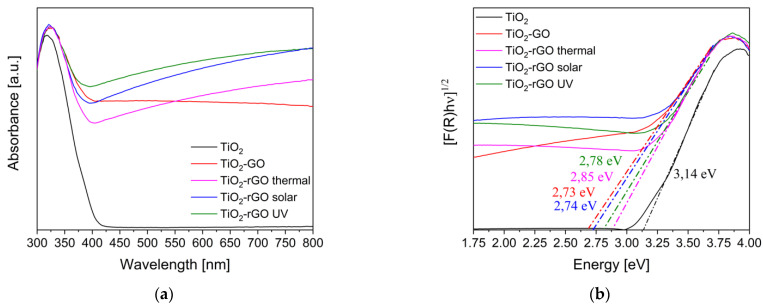
(**a**) UV-DRS spectra of the photocatalysts; (**b**) Kubelka-Munk functions for the optical band gap estimation.

The UV-vis DRS spectra of the materials and the corresponding Kubelka-Munk functions for the optical energy gap estimation are reported in the Figure 1a,b, respectively.

**Figure 2:**
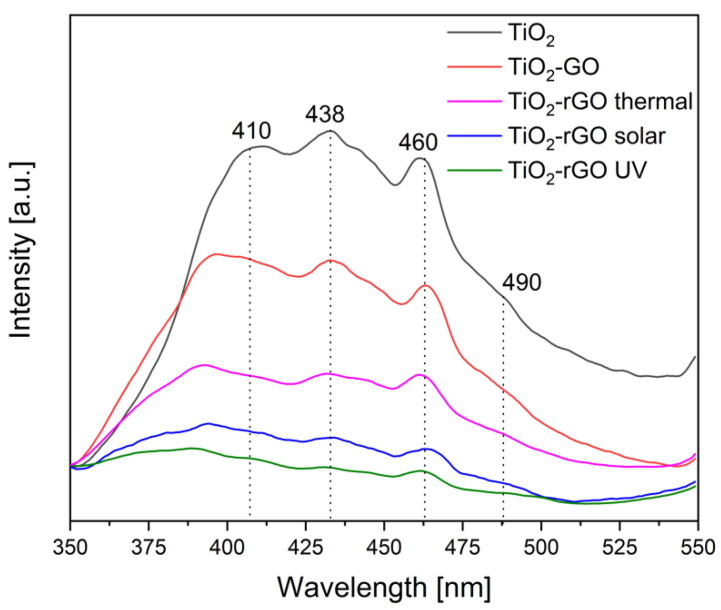
Photoluminescence spectra with λ_exc_ = 300 nm.

In comparison with the bare TiO_2_, the samples containing GO and rGO show a marked absorption on all visible range, stronger for the thermal treated and UV or solar reduced samples. Furthermore, the absorption band onset of the composites exhibits a slight red-shift probably due to the interaction between the titania surface and the graphene oxide functional groups [35]. As consequence, from the TAUC plot it is possible to note a decrease of the optical band gap from 3.15 eV of pure TiO_2_ to about 2.7–2.8 eV of the samples modified with GO and rGO, confirming as their addition on TiO_2_ improves the titania visible light absorption, as also reported in the literature [14,16]. Figure 2 depicts the photoluminescence spectra acquired with an excitation wavelength of 300 nm.

**Figure 3:**
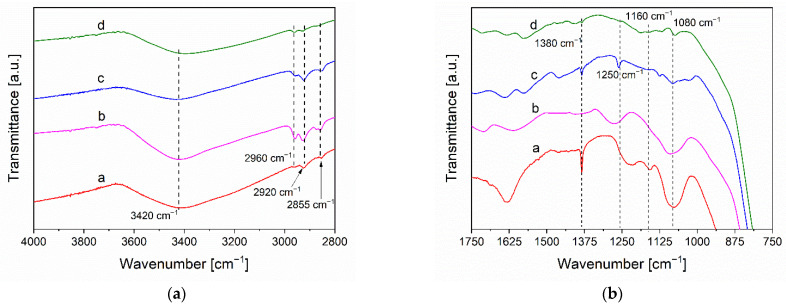
FTIR spectra of: a- TiO_2_-GO (red line) b- TiO_2_-rGO thermal (magenta line); c- TiO_2_-rGO solar (blue line) and d- TiO_2_-rGO UV (olive line). The spectra are separated in two graphs in order to facilitate the reading: (**a**) region from 4000 to 2750 cm^−1^, (**b**) region from 1750 to 750 cm^−1^.

It is possible to distinguish four different bands. The first one, which in bare TiO_2_ is located at around 410 nm, is due to band-to-band recombination. The second one, placed at 438 nm, is related to the excitons recombination, and the last two at 460 nm and 490 nm are due to trapped electrons and trapped holes defects, respectively [36]. In the TiO_2_-GO and TiO_2_-rGO based samples, there is a reduction of the bands emission intensity. This can be attributed to the presence of GO, which subtracts some electrons from the semiconductor surface and reduces the radiative recombination phenomena [37]. As reported, it is possible to estimate the reduction degree of GO from the relative intensity between the GO and rGO containing samples [38]. The TiO_2_-GO sample exhibits bands with higher intensity, compared to the TiO_2_-rGO based composites. This behavior well fits the higher conductivity of rGO respect to GO, which permits a better capture of electrons. Furthermore, considering the three reduced samples, it is possible to note as the composites treated with the UV or solar light show a lower intensity of bands compared to the sample prepared with the thermal treatment, pointing to a higher efficiency of the photoreduction of GO than the thermal route. FTIR spectra of the samples are investigated in the Figure 3.

The broad and intense signal at around 3420 cm^−1^ (Figure 3a), visible in all samples, corresponds to the O–H stretching of oxygenated groups [39]. The small characteristic peaks of graphene oxide at 2920 cm^−1^ and 2855 cm^−1^ are related to asymmetrical and symmetrical –CH stretching of methylene group, respectively [39]. It is interesting to note that the signal at 2960 cm^−1^, related to the asymmetrical –CH stretching of methyl group, is more intense in the TiO_2_-rGO based samples, indicating the occurred reduction of GO [39,40,41]. The Figure 3b shows the region between 1750 cm^−1^ and 750 cm^−1^ in which there are several signals associated to many oxygenated groups. As reported, the decrease in intensity of these signals is related to a partial reduction of GO [39,40,41]. Among these, there are the oxygenated groups such C–OH at around 1160 cm^−1^ or the epoxydic ones placed at 1380 cm^−1^, 1250 cm^−1^ and 1080 cm^−1^ [42]. It is possible to note as there is a decrease in the intensity of these bands in the TiO_2_-rGO-based samples, confirming the occurrence of the GO reduction. Interestingly, the bands intensity of the TiO_2_-rGO thermal sample is higher than TiO_2_-rGO solar and TiO_2_-rGO UV composites. This is another indication of the better reduction of GO obtained with the photoreduction versus the thermal treatments. Finally, the broader signal between 800 cm^−1^ and 500 cm^−1^ is related to the bending and stretching of O–Ti–O bonds [39]. 

**Figure 4:**
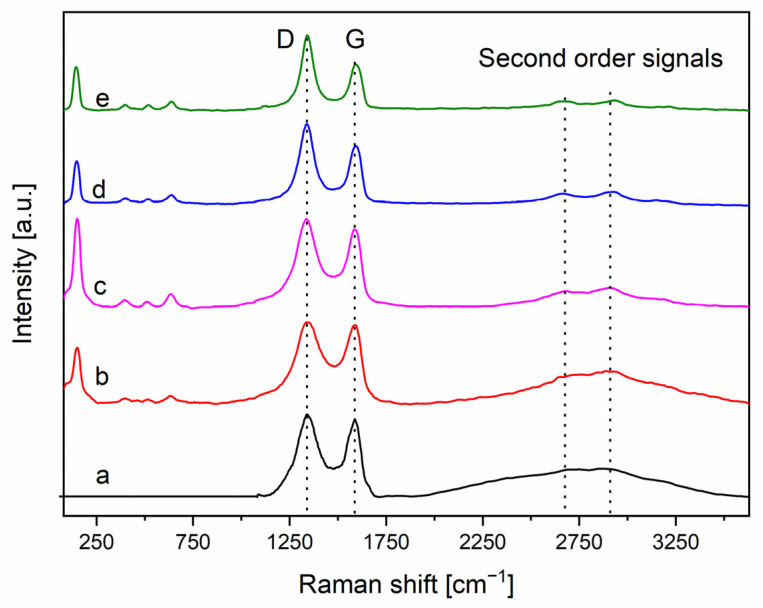
Raman spectra of the examined samples: black line- GO; red line - TiO_2_-GO; pink line - TiO_2_-rGO thermal; blue line - TiO_2_-rGO solar and green line -TiO_2_-rGO UV.

The Raman spectroscopy is a more sensitive technique to investigate the graphene oxide reduction. The spectra of the examined materials are illustrated in the Figure 4 and summarized in the Table 2.

At low Raman shift (150 cm^−1^, 397 cm^−1^, 518 cm^−1^, and 640 cm^−1^), there are the typical P25 peaks related to E_g_, B_1g_, A_1g_+B_1g_, and E_g_ vibrational modes, respectively. After the addition of GO and its reduction, these signals do not show any change in their positions or relative intensities, proving that there are no alterations in anatase and rutile phases structure [43,44]. 

In the GO, TiO_2_-GO and TiO_2_-rGO based-samples, the typical D and G bands (1360 cm^−1^ and 1600 cm^−1^ respectively) are present. The first one is related to out of plane vibrations of GO, often associated to crystalline structure disorder, whereas the second one is due to those in-plane, typical of hybridized sp^2^ carbons [45]. It is interesting to note how the I_D_/I_G_ signal ratio is similar for pristine GO and TiO_2_-GO samples, while is higher for the TiO_2_-rGO ones, highlighting a less ordered structure due to the partial reduction of sp^2^ bonds to sp^3^ lattice defects [45,46,47]. These additional defects can favor the titanium dioxide dispersion on the graphenic surface in addition to promoting a better interaction between them [45,48]. Another important parameter to establish the effective reduction of GO is the separation between D and G bands, often reported as FWHM (Full Width at Half Maximum) also considering the band position [49,50]. In the Table 2 are reported all the parameters obtained fitting a Lorentzian curve on each signal.

By the data of Table 2, it is clear that both the I_D_/I_G_ signal ratio and the FWHM of the peaks suggest a greater reduction degree for UV sample, followed by solar and then by thermal treated sample. Indeed, a lower FWHM value and a higher I_D_/I_G_ ratio, often mean a higher reduction degree for the rGO. Finally, the widening of the D peak in TiO_2_-GO respect to bare GO is a further proof of the interaction between the graphenic sheets and the titania nanoparticles.

**Figure 5:**
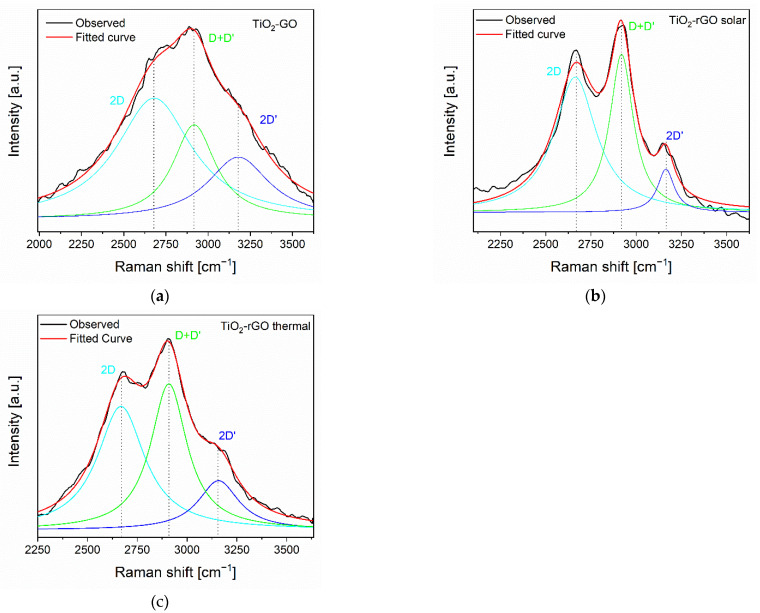
Second-order Raman signals for TiO_2_-GO (**a**), TiO_2_-rGO solar (**b**) and TiO_2_-rGO thermal (**c**). The spectra are acquired with an excitation wavelength of 532 nm.

To further examine the defects generated by the reduction of GO, the second order region was studied. In the Figure 5 reports a detailed investigation of the region between 2250 cm^−1^ and 3650 cm^−1^ for the TiO_2_-GO, TiO_2_-rGO solar, and TiO_2_-rGO thermal samples. The UV reduced sample is very similar to the solar treated, whereas the thermal treated exhibits an intermediate behavior between the photo-reduced TiO_2_-rGO samples and the unreduced TiO_2_-GO.

On the basis of the literature data, it is possible to note three principal signals [51]. The first one called 2D (or even G’) at around 2690 cm^−1^ is related to defective structures of GO. This signal is usually absent in defect-free structures like pure graphite [52]. The second band, coded as D+D’ at around 2900 cm^−1^, showed a more remarkable intensity variation in the examined samples. This band, as the 2D, is ascribed to the combination of phonons with different momenta, so the presence of defects is necessary to allow the transition according with the selection rules [52,53], and then this band can be correlated to a more defective structure. The higher intensity of the D+D’ band exhibited by TiO_2_-rGO solar is further evidence of the occurrence of a strong interaction between the TiO_2_ and rGO. Finally, the last band identified as 2D’ is associated to the second order of 2D signal that is permitted by selection rules and is not associated to defective structures [52,53].

### 3.2. Photodegradation Results

**Figure 6:**
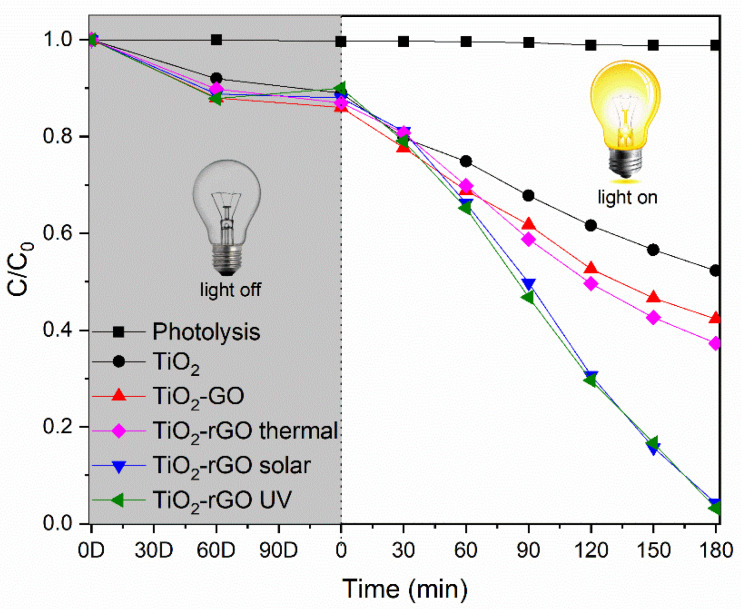
2,4-D photodegradation. The experiments were conducted three times with an error below 1 %, i.e., within the symbols thickness. The letter D after the firsts four x-values indicate the dark conditions; the time 0 is equal to 120 D.

The photodegradation measurements for the 2,4-D removal are reported in the Figure 6. A photolysis experiment without photocatalyst was carried out to establish the target molecule photostability. After three hours of irradiation, no change in concentration was detected, so the light source does not degrade the herbicide. 

During tests conducted in dark for five hours, all the samples reached the adsorption-desorption equilibrium after 120 min, stabilizing at around 10 % the contribution of the adsorption. No concentrations variations are detected after 2 h. 

Upon the irradiation, the bare TiO_2_ shows the worst degradation efficiency with a total removal of 48 % respect to the initial concentration. The inclusion of GO in bare titania improves its activity up to 58 %. These data confirm the synergistic effect of GO in light absorption and charge separation, that enhances the TiO_2_ photocatalysis. Reduced samples exhibit the greatest activity, due to the higher charge mobility induced by the rGO. Among these, the thermal treated sample shows the lowest photocatalytic improvement. This can be due to the lower reduction degree of GO in the TiO_2_-rGO thermal as stated by Raman and PL measurements, and to the lower surface area (44 m^2^·g^−1^ vs 53 and 55 m^2^·g^−1^ of the other two rGO based samples). The TiO_2_-rGO solar and TiO_2_-GO UV showed the best activity, with the 97 % removal of pollutant for both materials with a similar catalytic behavior. Considering that the synthesis of TiO_2_-rGO UV requires a UV source that is more expensive and less safe than the solar one, the sample preparation employing the solar source could be more attractive from an economical and environmental point of view.

Furthermore, the calculated kinetic constants for these photodegradations are reported in the Table 3. It is important to note, that the 2 wt.% of rGO was chosen as the best amount of promoters on TiO_2_. Indeed, a higher or a lower percentage of rGO, decreases the kinetic constant. (See supplementary materials, Table S1).

**Figure 7:**
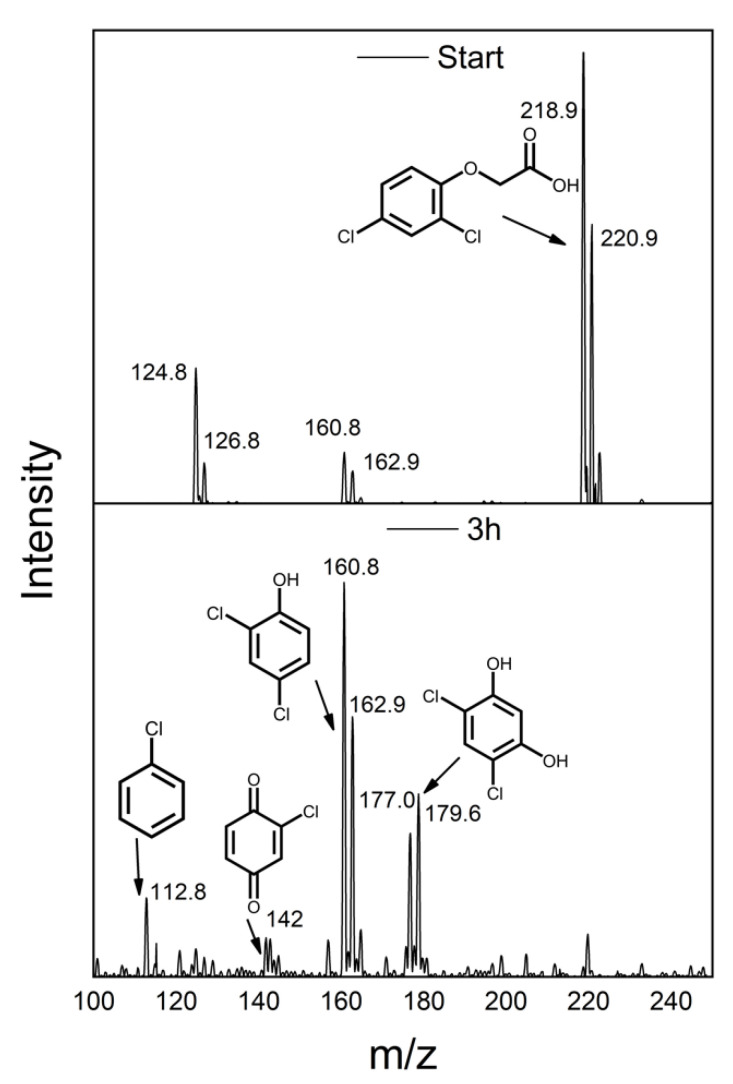
ISI-MS spectra acquired during the photocatalytic tests using the TiO_2_-rGO solar sample. In the upper panel the spectrum before the photocatalytic experiment, in the bottom after 3 h irradiation.

**Figure 8:**
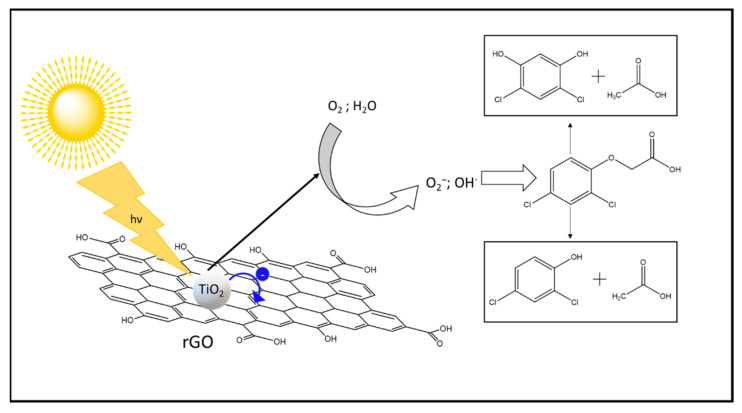
Proposed photocatalytic mechanism.

Figure 7 shows the ISI-MS spectra for the as prepared 2,4-D solution without catalyst (upper panel) and the centrifuged solution after 3 h irradiation in presence of TiO_2_-rGO solar (bottom panel), in order to investigate the by-products derived by photodegradation. It is possible to note by the first spectrum that the principal signals are related to the molecular peaks of 2,4-D, while the signals around 161 m/z and 125 m/z, are ascribed to 2,4-dichlorophenol and to 1,2,4-benzentriol, respectively, both deriving by fragmentation processes. After the irradiation, the molecular peak is present only in trace amounts, with a very low intense peak. The principal signals are assigned to 2,4-dichlorophenol (DCP, around 162 m/z) and to 2,4-dichlororesorcinol (DCR, 178 m/z), formed by attack of the radical OH to the alkyl chain or to the aromatic ring respectively [54,55]. Other less abundant signals are visible, related to 2-chlorobenzoquinone (142 m/z), probably formed after a subsequent radical attack to DCP, and chlorobenzene (113 m/z) [54,55]. Although these by-products are dangerous, it is important to highlight as the complete removal of a more persistent contaminant as the 2,4 D in water (even at low concentration) is a promising result, considering that these formed intermediates, present in trace, can be degraded more easily with further irradiation processes [3,49,50] (after another 3 h of solar irradiation no ISI-MS signals were detected). The TOC analyses on TiO_2_-rGO solar sample after 3 h of irradiation reported a value of 54%, suggesting a partial mineralization of 2,4-D, according to the literature on TiO_2_-based photodegradation of the 2,4-D [30,54]. A further solar irradiation of 3 h allowed to reach a value of 96% i.e., the total mineralization. These results indicate a probable mechanism based on the interaction between the hydroxy radical and the 2,4-D molecule, giving the DCP and DCR as main by-products after 3 h of solar irradiation, as confirmed by the ISI-MS results. The proposed photocatalytic mechanism is reported in the Figure 8. The solar irradiation allows to excite the TiO_2_ with the formation of the e^−^/h^+^ pairs. The photoelectrons are able to reduce the graphene oxide to obtain the rGO promoter, allowing at the same time an efficient charge carriers separation. Indeed, the e^−^ move on the GO layers limiting their recombination with the holes. Successively, the obtained material oxidizes the 2,4-D pesticide thanks to the generation of hydroxyl radicals (formed due to the interaction of e^−^/h^+^ with the oxygen and the water present in the reaction environment) with the formation of DCP and DCR as main by-products.

**Figure 9:**
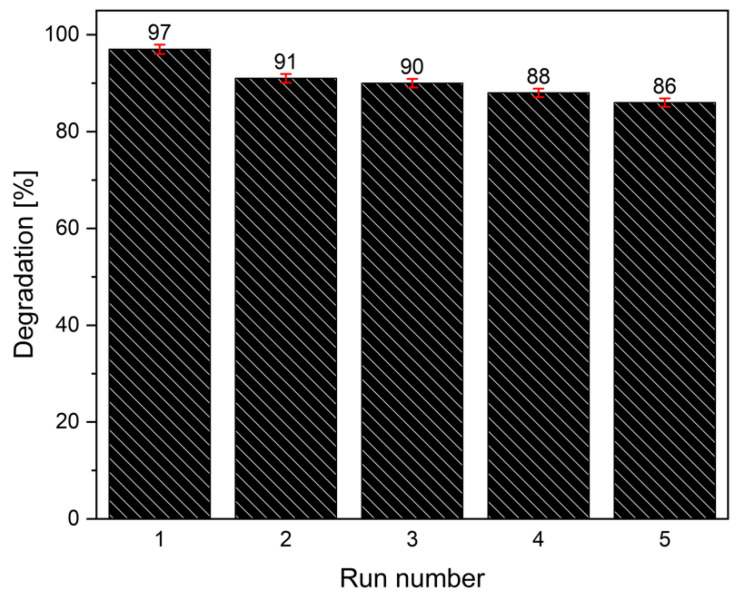
Reusability tests for TiO_2_-rGO solar sample in the 2,4 D photocatalytic degradation.

In order to verify the reusability of this catalyst, five successive measures were made with the same powders (Figure 9). After each experiment, the catalyst was centrifuged, washed, dried, and collected for the subsequent test.

From the histograms in the Figure 9, 11% decrease of activity can be seen, reasonably attributed to the loss of powders during washing operations (about 1 mg for each run). However, the pesticide degradation efficiency remained satisfactory being higher than 85%.

The Zeta potentials for the TiO_2_, TiO_2_-GO and TiO_2_-rGO solar samples were reported in the supporting information (Figure S7). It is possible to note as the bare TiO_2_ showed the highest values independently to the pH, whereas the addition of the carbonaceous promoters led to a decrease of the Zeta potentials with a similar behavior of the TiO_2_-GO and the TiO_2_-rGO solar samples. The presence of these species, indeed, modified the charge balance of TiO_2_, being these compounds mainly deposited on the surface of the titanium dioxide.

The influence of the pH on the photocatalytic activity of the best sample (TiO_2_-rGO solar) was examined adjusting the pH of the 2,4D solution with HCl and NaOH 1M. The results are reported in the Table 4. The as-prepared 2,4-D solution used for the photocatalytic tests had a pH of 3.5, that was also the condition in which we have obtained the best results with the TiO_2_-rGO solar sample (kinetic constant of 0.16·10^−3^·s^−1^). As showed in the Table 4, neutral or basic conditions were detrimental for the photocatalytic activity. These data can be explained considering the Zeta potential reported in the Figure S7. Indeed, at pH 3.5 the 2,4-D was in its anionic form whereas the TiO_2_-rGO solar sample showed a little positive potential; as a result, there was a good interaction between them. On the contrary, neutral or basic conditions did not influence the pollutant anionic form, but made negative the surface of the photocatalyst, so their interaction was worsened. At pH 9.5 it was obtained a kinetic constant (0.14 10^−3^·s^−1^) slightly higher than neutral pH (0.11 10^−3^·s^−1^). This can be related to the higher concentration of hydroxyl ions, source of OH radicals, whose formation was favored at basic pH. Stronger acidic or basic conditions were not investigated due to the difficulty in the nanoparticles separation from the reaction slurry.

In the Table 5 the as-obtained photocatalytic results were compared with those of other samples reported in the literature.

As it is possible to note, the sample TiO_2_-rGO solar of this work, reports the highest 2,4-D degradation percentage using a solar radiation mimic lamp, with the 97% of removal. The molecular imprinting approach described in [54] leads to a selective photodegradation of 2,4-D, but a less performing activity with the 75% of removal with an UV lamp in 4 h. The P25 nanobelts, prepared by Chenchana et al. [56], have obtained the highest percentage of degradation (99% after 2 h), but the employment of UV source and Au nanoparticles makes the system less convenient and more expensive. The Fe-doping approach proposed in [57] leads to a moderate removal of pollutant, considering the lower amount of photocatalyst employed. However, the light source used is considerably more powerful, so less cheap and sustainable. The work of Lima et al. [58] is the only one that does not use the titanium dioxide. Despite the good removal percentage (up to 91%) reached with few amounts of catalyst (0.4 g/L) the employment of UV-C lamp as source, could give some problems for a possible practical or industrial application. Finally, it must be underlined that our data were achieved with a one-pot preparation, using the same experimental setup (reactor, light source, stirring rpm, and temperature) employed for the degradation tests, a fascinating aspect that can favor large-scale applications.

### 3.3. Toxicity Tests

In order to assess the potential toxic effect of titanium dioxide-reduced graphene oxide (TiO_2_-rGO) nanocomposites, a short-term test was performed by exposing nauplii of microcrustacean *Artemia* sp. to solutions containing TiO_2_-rGO solar (the most promising sample). It is known that *Artemia* is a saltwater microcrustacean widely used as a model organism to evaluate the impact of many compounds [59,60]. It is a non-selective filter-feeder and nano or microparticles smaller than 50 µm can be adsorbed by nauplii at the first life stages [61]. The genus *Artemia* sp. has evolved a tolerance to more saline media and, of these, *Artemia salina* is the most successful, in fact it has a wide-spread distribution in salt pools and salt lakes of high salinities [62]. Due to the ease and the speed of reproduction inside a laboratory, the commercial availability of durable cysts and its widespread distribution [59], it is a good model organism also to assess nanotoxicity. The mortality percentages of immobilized nauplii, obtained after 24 and 48 h of exposure to TiO_2_-rGO at 1% and TiO_2_-rGO at 2% at different concentrations, are reported in the Table 6.

Our results showed low percentage of immobilization both for TiO_2_-1% rGO and TiO_2_-2% rGO after 24 and 48 h of exposure for all concentrations tested.

Statistical analysis by one-way ANOVA test did not show significant values nor for the percentages of immobilization of treated nauplii after 24 and 48 h of exposure nor between treated and control (Ctrl) groups (*p* > 0.05).

**Figure 10:**
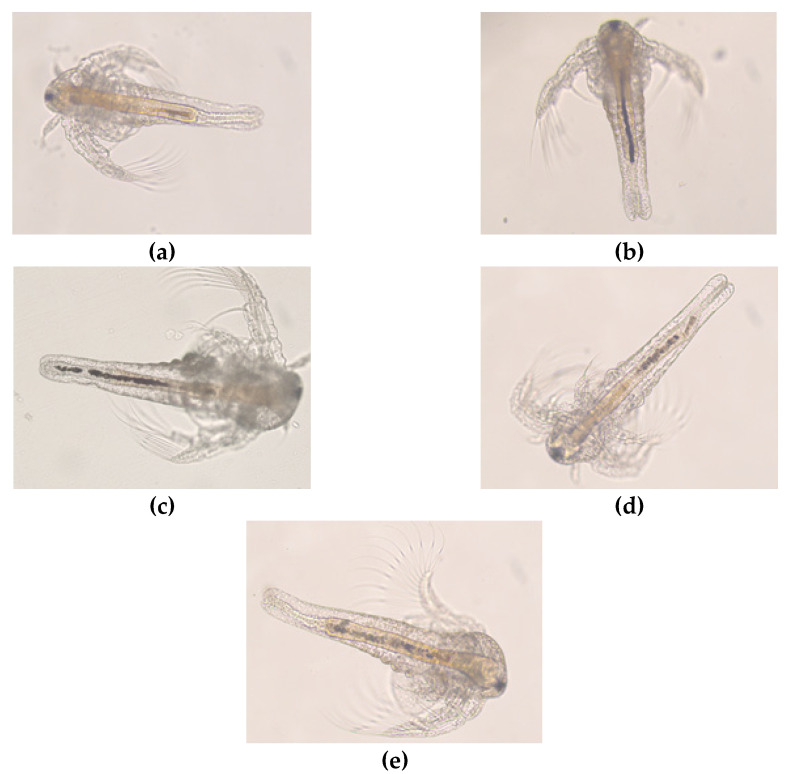
*Artemia nauplii*. Untreated (ctrl) (**a**); nauplii exposed to TiO_2_-rGO at 2% for 48 h at different concentration: 10^−1^ (**b**), 10^−2^ (**c**), 10^−3^ (**d**), 10^−4^ (**e**).

From the observed data, it is possible affirm that TiO_2_-rGO solar composites at the given concentrations, do not affect the vitality of the larval stage of the model organism; furthermore, no morphological and behavioral changes were found. It is particular to note that the gut was empty in the control (Figure 10a), while after exposure, nauplii started to ingest TiO_2_-rGO until the gut was almost filled evidenced by a dark line inside the gut after 48 h of exposure (Figure 10b,c,d,e).

Based on the results of mortality assay, Zhu et al. [63] showed that the acute exposure of Artemia salina larvae (instar I, II, and III) to GO induces significant changes in mortality and morphological and physiological parameters; however, some of the concentration used for treatments of GO were higher (respectively 25, 50, 100, 200, 400, 600 mg/L) than used in this study. On the contrary, in another study [64] a weak toxic effect of GO on Artemia franciscana was shown. The organisms at the adult stage were more sensitive than those at the nauplii instar I. Specifically, GO significantly increased the mortality of adults by 25% only at the highest concentration (100 μg/mL) and 72 h exposure; considering that the toxic effects of GO and rGO are observed only at very high concentrations (100 μg/mL) and long exposure times (72 h), the authors hypothesize a low ecotoxicological impact of the examined rGO on this target organism.

Finally, further studies on aquatic model organisms are needed to reach a better knowledge of the ecotoxicological impact of carbon-based nanocomposites.

## 4. Conclusions

The preparation of titanium dioxide-reduced graphene oxide composites was investigated, focusing the attention on the GO reduction method. Furthermore, the photodegradation of 2,4-D pesticide was carried out in order to investigate the photocatalytic properties of the prepared materials. From the obtained data, TiO_2_-rGO solar and TiO_2_-rGO UV gave the best results, both removing the 97% of pollutant from water. The oxidation of 2,4-D formed two principal degradation products, the 2,4-dichlorophenol (DCP) and the 2,4-dichlororesorcinol (DCR). Interestingly, the TiO_2_-rGO solar composite did not show substantial toxicity effects on the vitality of the microcrustacean *Artemia* sp. Furthermore, the obtained reduction degree for graphene oxide was greater in TiO_2_-rGO UV, followed by TiO_2_-rGO solar and lastly by TiO_2_-rGO thermal. These results are supported by Raman spectra, which shown this trend both in I_D_/I_G_ and in separation of signals, by the PL measurements which confirmed these data and by FTIR spectroscopy that showed a lower intensity of the bands related to the oxygenated moieties in the reduced samples. However, the one-pot synthesis by solar reduction is certainly the most promising preparation method thanks to the very good photocatalytic performances, the less expensive solar light employment instead of the UV, and finally the possibility to exploit the same irradiation source in the preparation and in the application of the photocatalysts.

## Figures and Tables

**Table 1:** Textural properties of the examined samples.

Photocatalyst	Isotherm	S_BET_^1^ (m^2^·g^−1^)	d_P_^1^ (nm)
TiO_2_	IV; H1	50	24
TiO_2_-GO	IV; H1	47	24
TiO_2_-rGO thermal	IV; H3	44	18
TiO_2_-rGO solar	IV; H2	53	6
TiO_2_-rGO UV	IV; H2	55	5

^1^ Surface area and mean pore size determined by BET and BJH methodologies, respectively.

**Table 2:** Raman signals related to D and G bands obtained by Lorentzian fitting.

Sample	Peak D	Peak G	I_D_/I_G_ ratio	FWHM_D_	FWHM_G_
GO	1341	1581	1.05	143.96 ± 2.1	82.88 ± 1.6
TiO_2_-GO	1338	1590	1.05	191.09 ± 4.1	90.42 ± 2.5
TiO_2_-rGO thermal	1335	1583	1.13	145.93 ± 1.8	80.52 ± 1.3
TiO_2_-rGO solar	1340	1589	1.28	95.73 ± 1.3	67.98 ± 1.1
TiO_2_-rGO UV	1341	1592	1.37	77.2 ± 1.2	65.30 ± 1.3

All the reported values are in cm^−1^.

**Table 3:** Kinetic constants for 2,4-D degradation.

Sample	Degradation (%)	Kinetic Constant ·10^3^ (s^−1^)	R^2^
TiO_2_	48	0.06 ± 0.01	0.985
TiO_2_-GO	55	0.08 ± 0.03	0.998
TiO_2_-rGO thermal	63	0.10 ± 0.01	0.979
TiO_2_-rGO solar	97	0.16 ± 0.02	0.988
TiO_2_-rGO UV	97	0.17 ± 0.02	0.981

**Table 4:** Kinetic constant for the degradation of 2,4-D related to TiO_2_-rGO solar in function of pH.

pH	Kinetic Constant 10^3^ [s^−1^]
3.5	0.16 ± 0.02
7.2	0.11 ± 0.03
9.5	0.14 ± 0.02

**Table 5:** Comparison amongst some recent literature data on photocatalytic 2,4-D degradation.

Sample	Experimental Setup	Degradation [%]	Main by-Products	References
TiO_2_-rGO solar 1 g/L	Artificial Solar irr., 10.7 mW/cm^2^, 3 h	97	DCP, DCR	This work
MI TiO_2_/2,4D 1 g/L	UV irr., 365 nm 12 mW/cm^2^, 4 h	75	DCP, DCR	[54]
TiO_2_ P25-Au 1 g/L	UV irr., 365 nm 9 mW/cm^2^, 2 h	99	DCP	[56]
Fe-doped TiO_2_ 0.4 g/L	Artificial Solar irr., 32 mW/cm^2^, 2 h	72	-	[57]
Fe_3_O_4_@WO_3_/SBA-15 0.4 g/L	UV irr., 254 nm, 20 W, 4 h	91	-	[58]

**Table 6:** Percentages of immobilization of nauplii exposed to TiO_2_-rGO solar at 1 wt.% and TiO_2_-rGO at 2 wt.% at various concentrations (mg rGO-TiO_2_/ ml ASPM water) for 24 h, 48 h and controls (CTRL).

Sample	CTRL	10^−1^	10^−2^	10^−3^	10^−4^
TiO_2_-1%rGO 24 h	0%	1.56 %	4.16%	5.73%	6.77%
TiO_2_-1%rGO 48 h	0.52%	2.60%	5.21%	7.81%	8.85%
TiO_2_-2%rGO 24 h	0%	2.08%	2.08%	4.69%	2.08%
TiO_2_-2%rGO 48 h	0.52%	5.73%	5.73%	6.77%	5.21%

## Data Availability

The data presented in this study are available on request from the corresponding author.

## Supplementary Materials

The following are available online at https://www.mdpi.com/article/10.3390/ma14205938/s1, Figure S1. Measured emission spectrum of the Osram Ultra Vitalux lamp. Figure S2 and S3: N_2_ adsorption-desorption isotherms and BJH pore size distribution for non-reduced samples; Figure S4 N_2_ adsorption-desorption isotherms and BJH pore size distribution for the solar samples with different percentage content of rGO; Figure S5. (a) Adsorption-desorption equilibrium in the first two hours. (b) Photocatalytic degradation after the reached equilibrium; Figure S6. SEM images of samples: TiO_2_-GO in (a) and (b) at 2K and 50K magnification, respectively; the same for TiO_2_-rGO solar in (c) and (d) respectively; Figure S7. Zeta potential of the main samples in function of pH. Table S1: Kinetic constants measured at different wt.% of rGO.
