# Supplementary material for: One-Pot Synthesis of TiO2-rGO Photocatalysts for the Degradation of Groundwater Pollutants
Source: Materials (Basel). 2021 Oct 10;14(20):5938. doi: 10.3390/ma14205938 (PMC8539955; doi:10.3390/ma14205938)
Supplement: Supplementary file 1 [file materials-14-05938-s001.zip › materials-1382524-SM-for-conversion.pdf]

materials

Supplementary Materials

One-Pot Synthesis of TiO_2-rGO Photocatalysts for the Degradation of Groundwater Pollutants

Stefano Andrea Balsamo [1], Roberto Fiorenza [1,*], Marcello Condorelli [1], Roberta Pecoraro [2], Maria Violetta Brundo [2], Francesca Lo Presti [1] and Salvatore Sciré [1]

[1] Department of Chemical Sciences, University of Catania, Viale A. Doria 6, 95125 Catania, Italy; stefano.balsamo@phd.unict.it (S.A.B.), marcello.condorelli@unict.it (M.C.); francesca.lopresti@phd.unict.it (F.L.P.); sscire@unict.it (S.S.)

[2] Department of Biological, Geological and Environmental Science, University of Catania, Via Androne 81, 95124 Catania, Italy; roberta.pecoraro@unict.it (R.P.); mvbrundo@unict.it (M.V.B.);

* Correspondence: rfiorenza@unict.it; Tel.: +39 0957385012

Supplementary Materials

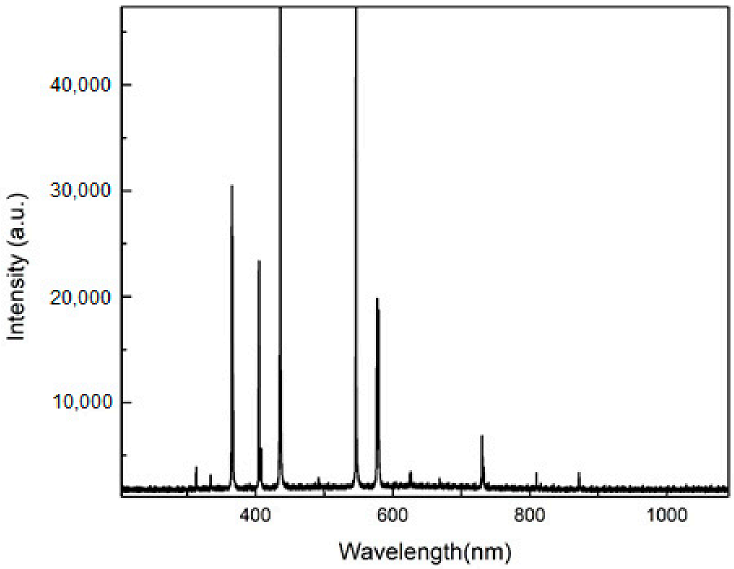

Figure S1. Measured emission spectrum of the Osram Ultra Vitalux lamp.

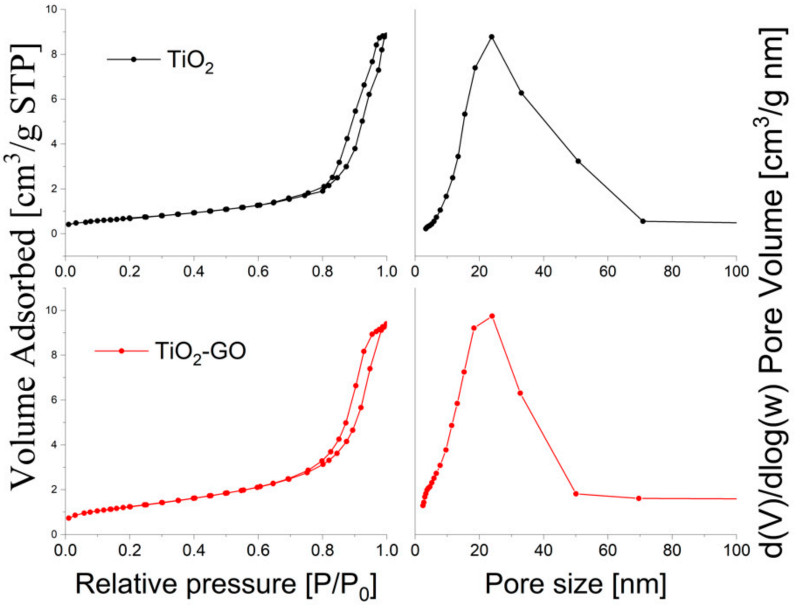

Figure S2. N_2 adsorption-desorption isotherms and BJH pore size distribution for the examined samples.

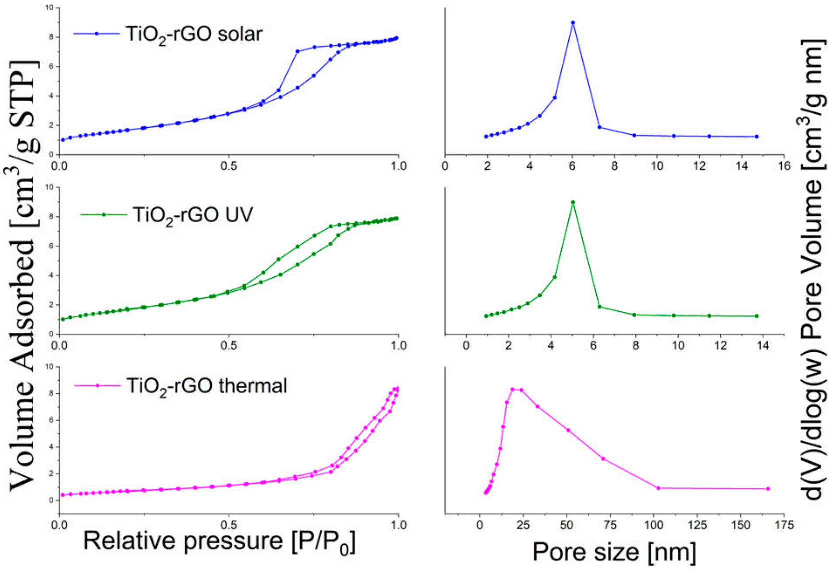

Figure S3. N_2 adsorption-desorption isotherms and BJH pore size distribution for the examined samples.

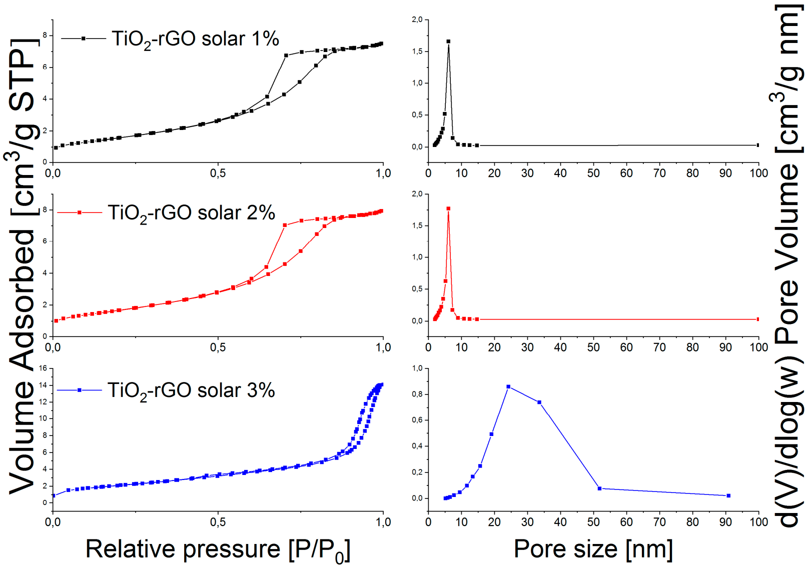

Figure S4. N_2 adsorption-desorption isotherms and BJH pore size distribution for the solar samples with different percentage content of rGO.

From the data shown in the Figure S4, it is possible to note how a small amount of rGO (1%, 2% wt) did not change the textural properties of the TiO_2-rGO composites. However, with the 3 wt.% of rGO, despite the surface area remaining similar, (S_{BET} = 51 $m^2 \cdot g^{-1}$, 53 $m^2 \cdot g^{-1}$ and 50 $m^2 \cdot g^{-1}$ for TiO_2-1%rGO solar , TiO_2-2%rGO solar, and TiO_2-3%rGO solar, respectively) the pore shapes and dimensions were different. The hysteresis changes from H2 to H3, related to slit-shaped pores, while the mean pores diameter increases from 6 nm of 2% sample to 16 nm of TiO_2-3%rGO solar, pointing to as the increase of the amount of rGO deposited on the surface of TiO_2 caused the pores' modification.

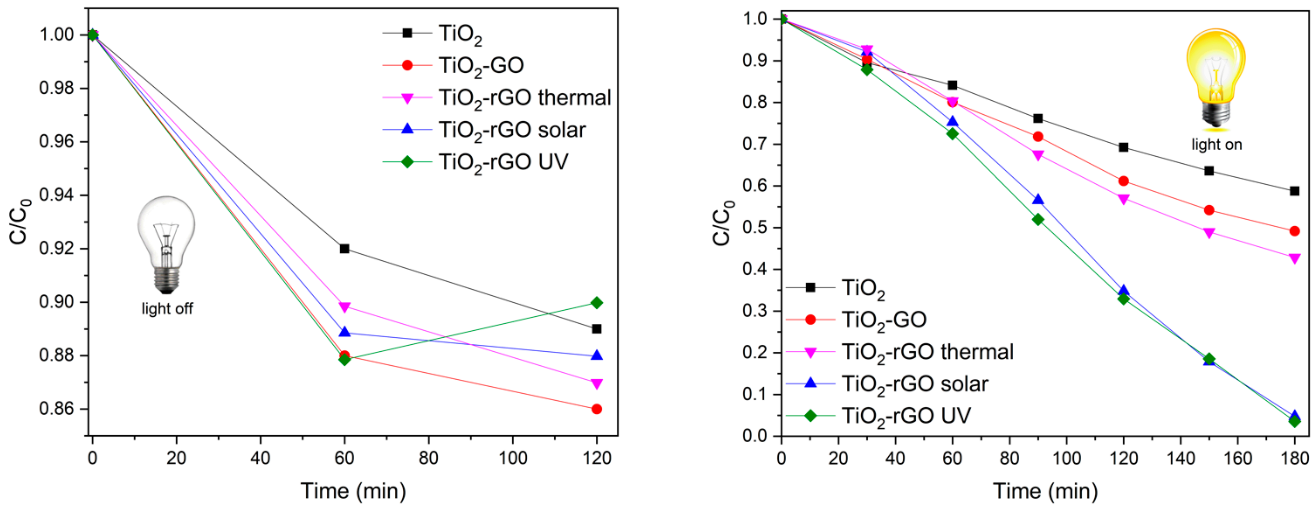

(**a**) (**b**)

Figure S5. (**a**) Adsorption-desorption equilibrium in the first two hours. (**b**) Photocatalytic degradation after the reached equilibrium.

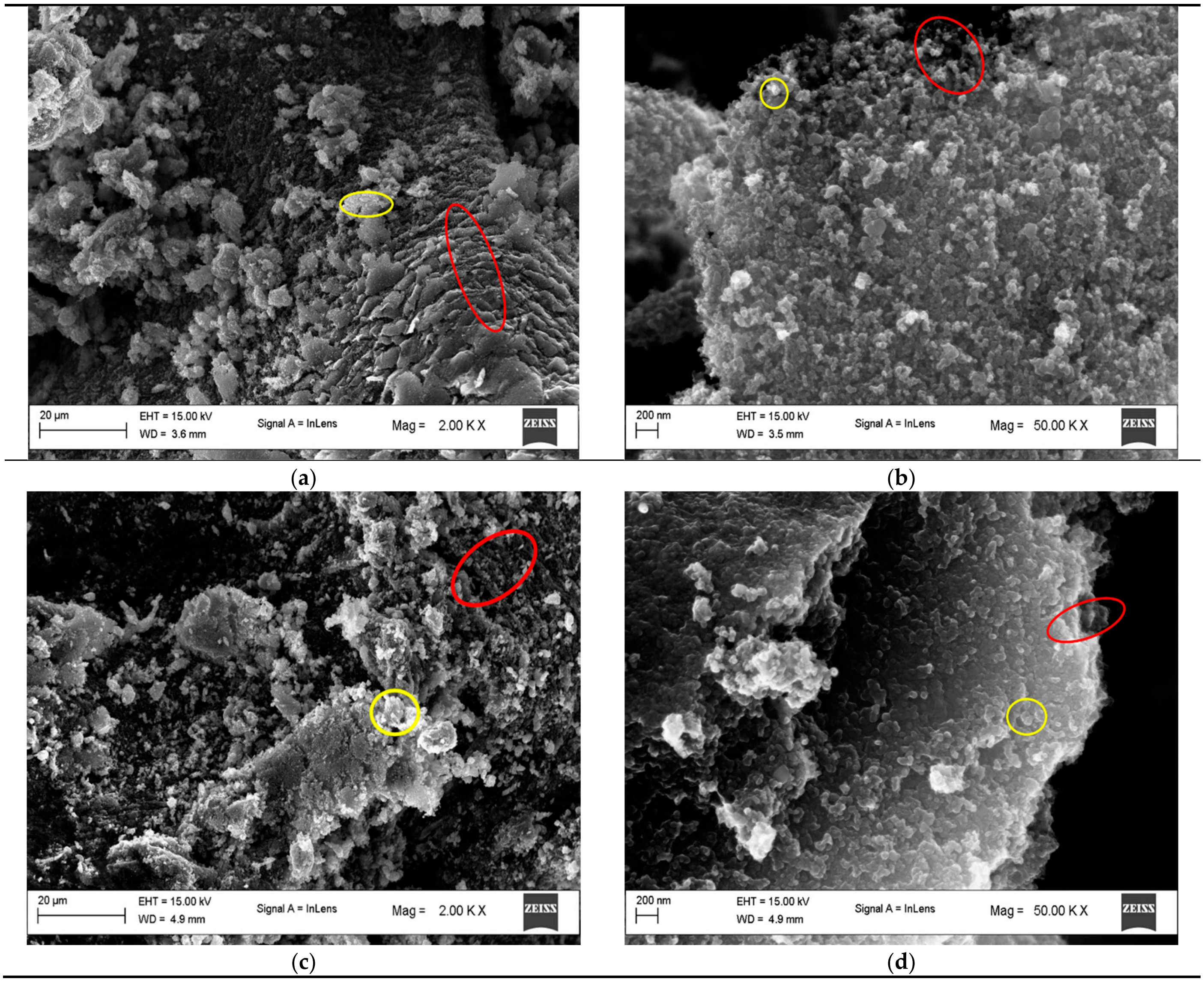

Figure S6. SEM images of TiO_2-GO (**a**) and (**b**) at 2K and 50K magnification respectively; the same for TiO_2-rGO solar in (**c**) and (**d**) respectively.

From the SEM images of TiO_2-GO and TiO_2-rGO solar, it is possible to distinguish the carbonaceous structures marked by the red circles, and the TiO_2 agglomerates by the yellow ones.

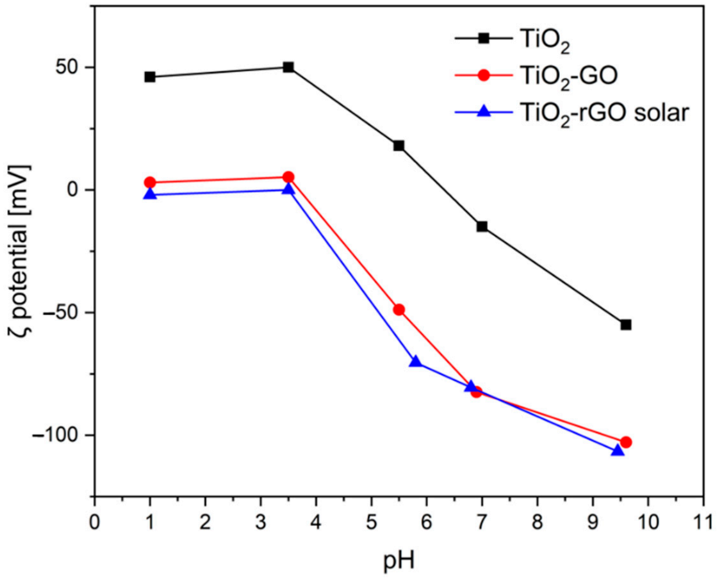

Figure S7. Zeta potential of the principal sample in function of pH.

Table S1. Kinetic constants measured at different wt.% of rGO.

wt.% of rGO	Total degradation (%)	Kinetic constant·10^3 (s^{-1})
TiO_2	48	0.06 ± 0.01
1%	80	0.13 ± 0.02
2 %	97	0.17 ± 0.01
3 %	90	0.15 ± 0.01

Consequently, the 2 wt.% was chosen as optimal content of rGO. Indeed, a lower amount reaches only the 80% of degradation, whereas a higher amount decreases the activity to 90% of degradation. The same considerations are similar considering the kinetic constants.
